# Selection on female reproductive schedules in the marula fly, *Ceratitis cosyra* (Diptera: Tephritidae) affects dietary optima for female reproductive traits but not lifespan

**DOI:** 10.3389/finsc.2023.1166753

**Published:** 2023-05-17

**Authors:** Kevin Malod, C. Ruth Archer, John Hunt, Susan W. Nicolson, Christopher W. Weldon

**Affiliations:** ^1^ Department of Zoology and Entomology, University of Pretoria, Hatfield, South Africa; ^2^ Institute for Evolutionary Ecology and Conservation Genomics, University of Ulm, Ulm, Germany; ^3^ School of Science, Western Sydney University, Penrith, NSW, Australia

**Keywords:** nutritional geometry, Tephritidae, life-history strategy, trade-off, lifespan, experimental selection

## Abstract

**Introduction:**

A changing environment can select on life-history traits and trade-offs in a myriad of ways. For example, global warming may shift phenology and thus the availability of host-plants. This may alter selection on survival and fertility schedules in herbivorous insects. If selection on life-histories changes, this may in turn select for altered nutrient intake, because the blend of nutrients organisms consume helps determine the expression of life-history traits. However, we lack empirical work testing whether shifts in the timing of oviposition alter nutrient intake and life-history strategies.

**Methods:**

We tested in the marula fruit fly, *Ceratitis cosyra*, how upward-selection on the age of female oviposition, in comparison with laboratory adapted control flies, affects the sex-specific relationship between protein and carbohydrate intake and life-history traits including lifespan, female lifetime egg production and daily egg production. We then determined the macronutrient ratio consumed when flies from each selection line and sex were allowed to self-regulate their intake.

**Results:**

Lifespan, lifetime egg production and daily egg production were optimised at similar protein to carbohydrate (P:C) ratios in flies from both selection lines. Likewise, females and males of both lines actively defended similar nutrient intake ratios (control =1:3.6 P:C; upward-selected = 1:3.2 P:C).

**Discussion:**

Our results are comparable to those in non-selected *C. cosyra*, where the optima for each trait and the self-selected protein to carbohydrate ratio observed were nearly identical. The nutrient blend that needs to be ingested for optimal expression of a given trait appeared to be well conserved across laboratory adapted and experimentally selected populations. These results suggest that in *C. cosyra*, nutritional requirements do not respond to a temporal change in oviposition substrate availability.

## Introduction

Seasonality is an important evolutionary driver because annual environmental fluctuations may select on life-history traits and trade-offs between them. In climatic regions where the year is usually characterised by favourable and unfavourable seasons (e.g., hot and cold or dry and wet), organisms have evolved strategies to cope with fluctuating environments and prioritise investment in some life-history traits over others, depending on the time of the year (1). In tropical and sub-tropical environments for example, where temperatures never drop to levels that may prompt diapause, insects shift their relative investment in reproduction versus survival as the seasons change (2–4). In such environments, the factor triggering this shift appears to be host plant availability. Hence, in *Anaea* butterflies (2) and *Bactrocera* fruit flies (3), populations prioritise survival over reproduction during the dry season, while populations invest in reproduction at the expense of life expectancy in the wet season. Furthermore, Clarke et al. (4) suggested that *Bactrocera* species undergo a reproductive arrest in the unfavourable season when host plants are unavailable or scarce. This differential seasonal investment in reproduction or survival that appears to be linked to host availability could be associated with a change in nutrient availability, or nutrient regulation, between seasons. Food sources used by adult tephritid fruit flies in nature are not well known, but consumption of fruit juice, nectar, faeces and honeydew is reported (5, 6). Supporting the idea of a differential seasonal investment linked to nutrition, individual nutrient intake affects ageing and reproduction in tephritid flies. Without a source of protein, which is essential to sexual maturation, flies survive in a “waiting mode” (7, 8). Accordingly, seasonal variation in nutrient availability could shift individual investment in reproduction versus survival. Similarly, organisms could adjust their nutrient regulation strategies to adjust expression of either trait. Understanding how variation in host availability affects nutrient regulation and life-history, is particularly important in a world where the phenology of herbivorous insects and their hosts is changing (9, 10).

The nutritional geometric framework (NGF) is a dietary mapping technique that allows us to determine how the intake of two or more nutrients, both singly and in combination, affects the expression of traits of interest (11). Typically, the NGF has been used to test how protein (P) and carbohydrate (C) affect phenotypes in herbivorous species, and P and lipid (L) in carnivorous species (12). The NGF is a powerful tool to study how these nutrients interact to shape life-history traits and the trade-offs between them. Using this approach, it was shown that expression of traits including lifespan (13–15), fertility (16) and immune function (17) depend critically on the ratio of nutrients that organisms consume.

Meta-analyses show that the negative impact of high P:C ratios on survival is widespread across Orthoptera, Hymenoptera, Diptera and Lepidoptera (18, 19). While high protein typically reduces lifespan, protein is often necessary for female fertility (20). This means that the nutrient blends that optimise female lifespan and fertility are often different. This necessitates a resource-based trade-off because females cannot eat a nutrient blend that maximises expression of both traits at the same time. Our understanding of dietary optima for male reproduction is far less developed than our understanding of female dietary requirements. Nevertheless, in species for which we have data in both sexes, typically females experience a diet-mediated trade-off between lifespan and fertility, while males do not (14, 21).

When the optimal nutrient blends for lifespan and fertility differ such that individuals cannot optimise both traits simultaneously, individuals given a choice of foods can regulate their intake to maximise expression of either trait or self-select an intermediate nutrient blend that allows moderate expression of both traits. In *Ceratitis cosyra*, females offered a choice of diets choose a nutrient blend (1:3 P:C) closer to the optimum for daily egg production (1:2.5 P:C) than lifespan (0:1 P:C) (15). However, in field crickets, females appear to choose a nutrient blend (1:2.2 P:C) that is intermediate between the dietary optima for lifespan (1:8 P:C) and daily fecundity (1:1 P:C) (16). Hence, flexible dietary intake allows individuals to prioritise specific life-history traits and adjust their life-history strategies. While different species appear to resolve trade-offs in different ways, it is unclear how often strategies of nutrient intake differ within species, between the sexes or among different populations.

In nature, life-history strategies vary enormously between populations. For example, lifespan can differ within species along latitudinal or altitudinal gradients (22) or in relation to the number of predators that occur in sympatry (23). It is not clear if this affects how individuals regulate their nutrition in species that experience dietary mediated trade-offs between lifespan and fertility. In populations under selection for long lifespan, individuals might regulate their nutrient intake towards nutrient blends that promote lifespan. Similarly, if populations are under selection for a shorter lifespan, it could be expected that individuals regulate their intake towards nutrient blends that maximise short-term reproductive investment. To date, these ideas have not been tested empirically. Nevertheless, a modelling approach combining the NGF with an agent-based model predicts that intake of nutrient blends that favour reproduction at the expense of lifespan should evolve in conditions of increased mortality risk in adults (24).

To understand how the variation in host availability shapes the relationship between food, sex and death and strategies of nutrient regulation, we selected upwards on age of female reproduction in replicate lines of *C. cosyra*. After 20 generations of selection, these upward-selected lines showed delayed reproductive effort illustrating successful selection but, against expectations, lived shorter lives and males transferred fewer sperm at mating (25). In this species, females and males have divergent dietary optima for lifespan (females: 0:1 P:C; males: 1:10 P:C), and the best nutrient blend for fertility and survival differs in females (fertility: 1:2.5 P:C; lifespan: 0:1 P:C), but both sexes regulate their intake towards a 1:3 P:C ratio (15). The fitness consequences of this are unknown in males because dietary optima for reproductive success have not been determined, but in females, this nutrient regulation strategy improves daily egg production at the expense of reduced lifespan. Given the effects of the selection regime on the phenotype of the flies (i.e. upward-selected flies), we predict that the dietary optima for lifespan and reproductive traits changed in upward-selected lines but not self-regulated intake. As the selection regime also reduced male lifespan in a similar way, we predict that males will target a nutrient blend that does not differ from that of the females, and that female and male lifespan are optimised on very similar P:C ratios.

## Materials and methods

### Fly populations and husbandry

Infested mangoes from across the Mpumalanga province, South Africa, were collected and pupae of *C. cosyra* retrieved. The wild flies emerging from these pupae were used to establish a culture. The procedure followed to establish a culture of *C. cosyra* was identical to the one used in Malod et al. (25). In brief, the culture was maintained at ~ 23°C in a climate room with a 14:10 light:dark photoperiod. Adults were kept in groups of ca. 200 flies in 5 L plastic cages with food (hydrolysed yeast and sugar in separate dishes) and water (water-soaked cotton wool) *ad libitum*.

### Selection regime

From the culture, we established four replicate populations of two selection lines. In control (CT) lines, oviposition substrate was provided when flies were 15 days old, which is the average age when eggs are typically collected from this species in laboratory conditions (e.g., 5, 26). In upward-selected lines (US), oviposition substrate was provided when flies were 25 days old, meaning that only females that survived to this age to oviposit would contribute to the next generation. Because female *C. cosyra* do not store sperm for long periods of time (important decline in sperm storage observed at 14 days post-mating) (27), the effect of this selection regime on male fertility is likely to be similar to what is observed in females. We maintained the selection regime for over 35 generations. Experimental flies used in Experiment I were collected from the 35^th^ generation and flies tested in Experiment II were from the 37^th^ generation. Because flies were selected on age of oviposition, each selection regime was inevitably assayed at different time points. Therefore, selection lines differ in their assay date. However, given that results are similar across selection regimes and that the patterns of expression for lifespan and reproduction correspond with what was observed in previous generations (25, 28), it is unlikely that results were affected by a temporal blocking effect.

### Experimental diets and consumption

Fifteen liquid experimental diets (**Table S1**) were created that varied in their P:C ratios (0:1; 1:8; 1:4; 1:2; 1:1 P:C) and total concentration of protein and carbohydrate (45, 180 and 360 g/L). This diet range is similar to the one previously used for *C. cosyra* (15), except that we discarded the 2:1 P:C ratios due to the short lives of the flies on this diet (about 20 days on average), which indicates a pathological effect. A blend of 18 amino acids was used as a source of protein and sucrose as a source of carbohydrate (**Table S2**). All diets were prepared with equal concentrations of micronutrients (**Table S2**) and 1.3 mL/L of blue food dye (Robertsons, Johannesburg, South Africa) was added to facilitate reading of the liquid volume. Either one (Experiment I, no-choice experiment) or two (Experiment II, choice experiment) diets and water were provided to individual flies on their day of emergence in 200 µL pipette tips (ROLL s.a.s, Italy), capped loosely with putty-like adhesive (Bostik, South Africa).

The volume of food consumed was determined by measuring pipette tips containing liquid diets with 1 mm scale graph paper (Canson, France). We replaced pipette tips for food and water every 5 days (no-choice experiment), 4 days (choice experiment) or earlier if depleted. Food was measured before and after replacing the pipette tips. Consumption was calculated using the difference between the initial length (for 100 µL of liquid) and the remaining length of diet in the pipette tip. Linear measurements of consumption were converted into volumes with a mathematical function that was obtained from a standard curve (**Figure S1**). In the no-choice experiment, each diet had three pipette tips used as controls (i.e., placed in the climate room, but in containers without flies) to assess evaporation in the climate room. In the choice experiment, two containers with two pipette tips per diet were maintained in the climate room to assess evaporative loss. For both experiments, the volume of evaporated diet was measured at the same time as the volume of diet consumed by the flies. The amount of diet volume lost for each ratio and concentration was then used to correct consumption for evaporation.

### Experiment I: effects of five protein to carbohydrate ratios at three concentrations

For each line and replicate, we analysed the effect of protein and carbohydrate on lifespan (LS) and reproduction by providing one of the 15 experimental diets to each of 5 females and 5 males (n = 15 diets × 2 selection lines × 4 replicates × 2 sexes × 5 flies = 1200 flies). Virgin females and males were placed individually in plastic transparent containers (125 mL) within 24 h of emergence. Each fly was supplied with two 200 µL pipette tips, one containing filtered water and one containing 100 µL of experimental diet. Mortality, diet and water levels were checked daily. Reproductive effort was measured by recording female fecundity. To do this, at the base of the females’ containers we placed a black screw-top lid (ø 32 mm) as an oviposition dish, filled with 2.5 mL of 10% orange essence solution (Robertsons, Johannesburg, South Africa). The dish was covered with a double layer of laboratory film that was pierced several times. In this species, females are able to lay eggs even when they are virgin (26). The dish was placed for both selected lines from the beginning of the experiments, and eggs were counted every six days when dishes were replaced. These data allowed us to estimate daily egg production (eggs/day, DEP) for an individual, and lifetime egg production (i.e., giving the number of eggs laid throughout the entire lifespan, LEP). The average temperature and relative humidity (RH) in the climate room during the no-choice experiment was 24.2 ± 1.4°C and 55 ± 9% for the CT line and 22.9 ± 1.6°C and 58 ± 12% for the US line.

### Experiment II: nutrient intake under dietary choice

To assess how individuals regulate their nutrient intake when they have a choice of diets, we recorded consumption of protein and carbohydrate when flies were given a dietary choice using established methods and combinations of P:C ratios (14, 15, 21). Flies were maintained as in the no-choice experiment, the only difference being that instead of one diet, they were given a pair of diets. Flies were randomly assigned to one of the following dietary pairs (**Figure S2**): **Pair 1**: 1:1 (180 g/L) vs 0:1 (180 g/L); **Pair 2**: 1:1 (180 g/L) vs 0:1 (360 g/L); **Pair 3**: 1:1 (360 g/L) vs 0:1 (180 g/L); **Pair 4**: 1:1 (360 g/L) vs 0:1 (360 g/L). For each diet in the pair, 100 µL was provided in a different pipette tip. The diet pairs were tested for each selected line and replicate on five flies of each sex (n _total_= 280 flies). Due to one replicate line collapsing, the US line was tested on only three replicates in Experiment II. Diet consumption was recorded every four days, starting within 24 h after emergence over a period of 16 days. No flies died or escaped before the end of the experiment. The average temperature and RH during the assay for the CT line was 22.6 ± 2.1°C and 65 ± 7%, and during the US line assay 21.7 ± 1.2°C and 55 ± 4%.

### Statistical analyses

#### Experiment I

In the first experiment, as longevity among the diet groups was highly variable, we divided total consumption by days lived to express male and female consumption in mg per day so that consumption by individuals was more comparable. Moreover, to standardize the response variables (LS, LEP and DEP) and nutrient intake, a Z-transformation to a mean of zero and standard deviation of one was used. Data from flies escaping during the experiment or dying from non-natural death (trapped in a drop of liquid diet) were removed. Then, the statistical procedure described in detail in Rapkin et al. (29) and Bunning et al. (30) was followed. To summarise, a multivariate response-surface approach was used to estimate the linear and non-linear (interactions between P × P, C × C and P × C) effects of protein and carbohydrate on response variables (LS, LEP, DEP) for each selected line and sex. First, a model containing only protein and carbohydrate was built to assess if intake of each nutrient significantly affected each response variable. Then, a second model including the linear and quadratic and correlational effects of protein and carbohydrate was built to determine the non-linear and interaction effects on the response variable. Replicate was added to the models as a random factor (four levels) to account for replication. A sequential approach was then used to compare nutritional landscapes across selected lines in females and males, between sexes for LS, and across the different traits in females (LS, LEP and DEP). For these comparisons it was necessary to create a dummy variable “trait type” (i.e., a column in the dataset with the two compared traits). The landscape comparisons were also performed using a sequential model building approach. The first model contained the linear effects only and was compared using partial F-tests to a second model with the linear effects plus their interaction with the dummy variable. In the third model, the quadratic effects (P × P and C × C) were added, and the model was compared to a fourth model including the interactions with the dummy variable. Finally, the correlational effect (P × C) was added to the fifth model and compared to the final model including the interaction between the correlational effect and the dummy variable. When an overall significant difference between models was detected using the sequential approach, a univariate analysis was used to determine which nutrient contributed to the effect. Generalised linear mixed effects models were built using the glmmTMB function from the glmmTMB package (31). To visualize the data, nutritional landscapes were constructed with the function Tps from the package FIELDS (32) in R v 4.2.1 statistical environment (R core team, 2022, Vienna, Austria); untransformed values (raw data) for the response variables and nutrient intake were used to construct the surface responses. To determine the nutritional optima (i.e., the exact amount of protein and carbohydrate needed to optimise the trait) on these landscapes we used the function OptRegionTps from the OptimaRegion package (33). To test for any difference between lines in overall food consumption, we calculated the total consumption (P+C). A generalised linear mixed effects model was used, with total consumption (square-root transformation to normalise the residuals of the model) as the response variable, selection, sex and their interaction as fixed factors, concentration and P:C ratio as covariates and replicate as a random factor. The model was built using the glmmTMB function.

#### Experiment II

In the choice experiment, the intake of nutrients expected had individuals fed at random from each diet was calculated for each fly (i.e., the volume of nutrients consumed if flies ate equal amounts of each food in the diet pair). A random intake would indicate that flies were not regulating their nutrient consumption. Expected intakes were subtracted from the observed intake values and the difference was compared with zero (one sample t test). Having determined if flies eat at random or not on the diet pairs, we characterised nutrient regulation strategies (i.e., if there was a preference for protein or carbohydrate) (see supplementary material **Tables S4 and S5**). For each selection regime, sex and diet pair we calculated a cumulative intake for protein and carbohydrate, then a regulated intake point as the mean total intake of protein and carbohydrate across all diet pairs. To determine how the regulated intake point differed between selection regimes and between sexes within each selection regime, we ran generalised linear mixed models. In each model, carbohydrate intake was added as response variable, protein intake as a covariate, selection regime or sex as fixed effects, as well as their interaction with the covariate, and replicate was added as a random factor. A significant interaction between the protein intake and the fixed factor would indicate that the regulated intake point differs between selection regimes or sexes. Protein and carbohydrate intake was expressed in mg rather than mg per day as the dietary choice experiment was performed over the same duration of 16 days for all individuals.

## Results

### Experiment I: effects of five protein to carbohydrate ratios at three concentrations

**Figure 1:**
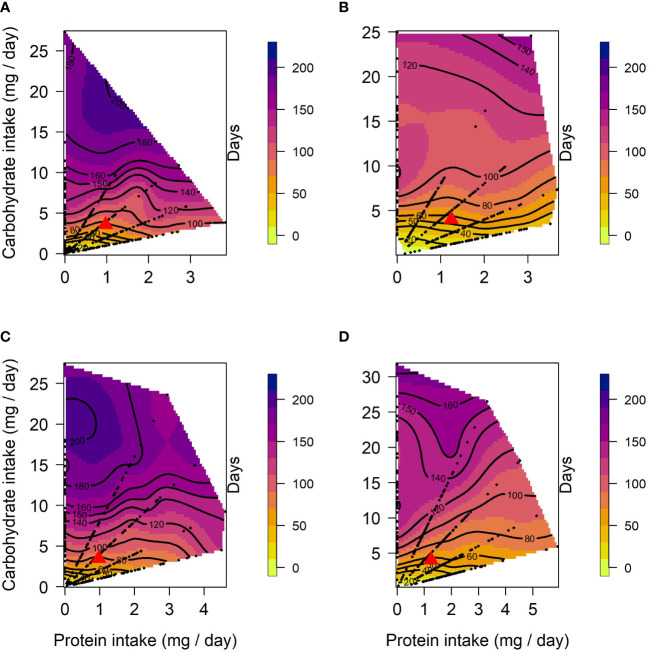
Nutritional landscapes for female **(A, B)** and male **(C, D)** lifespan in CT **(A, C)** and US **(B, D)** lines of *C. cosyra* selected on age of female oviposition. The colour gradient indicates how individuals perform for lifespan on a specific P:C intake. Red triangles indicate the regulated intake point. Each nutritional landscape represents 300 individuals.

In both selection lines, female lifespan (**Figures 1A, B**) peaked in the low protein, high carbohydrate region of the nutritional landscapes. However, the optimum for lifespan was more protein biased for US (Protein = 2.8 mg/day; Carbohydrate = 24.9 mg/day) than for CT females (Protein = 0.21 mg/day; Carbohydrate = 24.2 mg/day). These positive effects of carbohydrate on lifespan were evident in both selection lines as significant linear effects of carbohydrate (**Table 1**). There were also positive linear effects of protein intake on lifespan. However, in both cases lifespan increased more steeply with carbohydrate intake than protein intake (see gradients in **Table 1**). Further, in females of both selection lines, the significant negative quadratic effect of carbohydrate on lifespan indicated a peak in expression in flies fed a high carbohydrate diet (between 15 and 24 mg/day for CT and 23 and 25 mg/day for US). In addition, a significant positive quadratic effect of protein was detected in CT females, indicating a trough (1:30 to 1:10 P:C) ranging from low to high protein intake on the surface response (**Figure 1A**). Because the contribution of carbohydrate intake to lifespan was much greater than the contribution of protein, there was also a significant negative correlational effect, meaning that there was negative covariance between protein and carbohydrate that increased lifespan. No significant quadratic effect of protein or a correlational effect were detected in US females.

**Table 1:** Effects of five protein to carbohydrate ratios at three concentrations on lifespan (LS) and female reproductive traits (LEP and DEP) in *C. cosyra* in control lines (CT) and lines selected upwards on age of female oviposition for 35 generations.

Response variables	Linear effects		Nonlinear effects		
	P	C	P × P	C × C	P × C
Males					
Lifespan CT					
*Coefficient ± SE*	0.06 ± 0.03	0.81 ± 0.03	-0.02 ± 0.03	-0.18 ± 0.02	-0.11 ± 0.03
*χ2*	5.06	914.03	0.73	54.33	16.14
*p value*	**0.024**	**< 0.001**	0.392	**< 0.001**	**< 0.001**
Lifespan US					
*Coefficient ± SE*	-0.006 ± 0.03	0.84 ± 0.04	0.01 ± 0.03	-0.26 ± 0.03	-0.10 ± 0.04
*χ2*	0.03	407.62	0.11	52.32	7.40
*p value*	0.857	**< 0.001**	0.743	**< 0.001**	**0.006**
Females					
Lifespan CT					
*Coefficient ± SE*	0.15 ± 0.03	0.92 ± 0.03	0.09 ± 0.02	-0.21 ± 0.02	-0.10 ± 0.03
*χ2*	27.51	1199.84	10.98	83.97	14.60
*p value*	**< 0.001**	**< 0.001**	**< 0.001**	**< 0.001**	**< 0.001**
Lifespan US					
*Coefficient ± SE*	0.09 ± 0.03	0.81 ± 0.04	-0.003 ± 0.03	-0.17 ± 0.03	-0.07 ± 0.04
*χ2*	7.06	412.05	0.01	25.56	3.70
*p value*	**0.008**	**< 0.001**	0.931	**< 0.001**	0.054
LEP CT					
*Coefficient ± SE*	0.26 ± 0.05	0.55 ± 0.04	-0.01 ± 0.05	-0.16 ± 0.04	-0.08 ± 0.05
*χ2*	29.77	159.51	0.11	12.81	2.44
*p value*	**< 0.001**	**< 0.001**	0.74	**< 0.001**	0.741
LEP US					
*Coefficient ± SE*	0.18 ± 0.05	0.43 ± 0.06	0.02 ± 0.05	-0.05 ± 0.05	0.02 ± 0.06
*χ2*	11.41	50.64	0.16	0.95	0.10
*p value*	**< 0.001**	**< 0.001**	0.688	0.328	0.753
DEP CT					
*Coefficient ± SE*	0.22 ± 0.05	0.21 ± 0.05	-0.12 ± 0.06	-0.10 ± 0.05	-0.03 ± 0.06
*χ2*	16.52	16.61	4.32	3.53	0.32
*p value*	**< 0.001**	**< 0.001**	**0.037**	0.060	0.569
DEP US					
*Coefficient ± SE*	0.16 ± 0.06	0.02 ± 0.07	-0.04 ± 0.06	-0.02 ± 0.06	0.05 ± 0.07
*χ2*	7.20	0.10	0.40	0.10	0.53
*p value*	**0.007**	0.752	0.528	0.752	0.465

The analyses were run on results obtained from Experiment I where diets were provided under no-choice conditions. Bold values indicate significant effects (p<0.05).

In males, lifespan increased with intake of carbohydrate in both lines (**Figures 1C, D**), as indicated by a significant linear effect of carbohydrate (**Table 1**). A positive significant linear effect of protein was also found in CT males, indicating that lifespan also increased with the intake of protein, but to a lesser extent than carbohydrate (**Table 1**). As in females, there was a negative quadratic effect of carbohydrate in both lines (**Table 1**), indicating a peak in lifespan when carbohydrate intake was around 15 to 25 mg/day and 29 to 32 mg/day for CT and US respectively. The surface responses indicated that lifespan was optimised at low P:C ratios (**Figures 1C, D**). A negative correlational effect was detected in males of both lines, once again indicating a negative covariance between both nutrients that affect lifespan. For both CT and US males, lifespan was optimised at low P:C, the calculated nutritional optima were: Protein = 0.07 mg/day, Carbohydrate = 27.3 mg/day for CT, and Protein = 0.09 mg/day, Carbohydrate = 31.7 mg/day for US.

**Figure 2:**
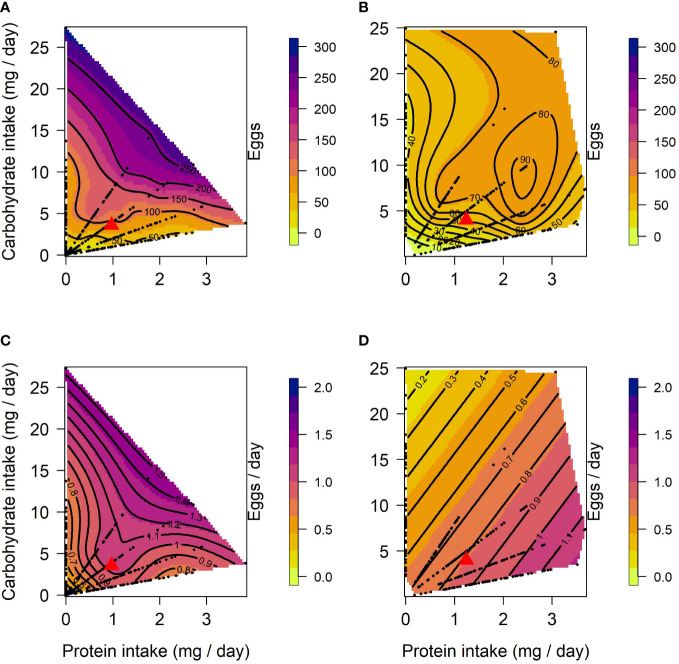
Nutritional landscapes for female lifetime egg production **(A, B)** and daily egg production **(C, D)** in CT **(A, C)** and US **(B, D)** lines of *C. cosyra* selected on age of female oviposition. The colour gradient indicates how individuals perform for a trait on a specific P:C intake. Each nutritional landscape represents 300 individuals.

The effects of protein and carbohydrate on female lifetime egg production were similar to those on lifespan. In both lines, there was a significant positive linear effect of protein and carbohydrate, with lifetime egg production increasing with the intake of both, but to a lesser degree with protein intake (**Table 1**). A significant negative quadratic effect for carbohydrate was only found in CT females, indicating a peak in the expression of lifetime egg production with carbohydrate intake around 24 to 27 mg/day. Optimal expression of lifetime egg production in CT females was at a higher carbohydrate intake relative to protein, the peak for lifetime egg production was determined to be at a protein intake of 0.01 mg/day and carbohydrate intake of 25.8 mg/day (**Figure 2A**). In contrast, there was no clear peak detected in US females for lifetime egg production (**Figure 2B**). No correlational effects were found in either line. Daily egg production for CT and US lines responded differently to dietary manipulations. In CT females, both protein and carbohydrate positively contributed to daily egg production expression to a similar extent (**Table 1**), and a significant negative quadratic effect of protein indicated a peak. Calculation of the nutritional optima indicated that the peak in daily egg production among CT females was at a protein intake of 1.6 mg/day and carbohydrate intake of 16.7 mg/day (**Figure 2C**). In contrast, only a significant linear effect of protein was detected in US females, indicating that an increase in protein intake contributed to higher daily egg production expression without any optimal value being reached in our study (**Table 1**) (**Figure 2D**).

Using a sequential building approach to compare nutritional landscapes for lifespan between selection lines, we found no difference in linear, quadratic or correlational effects of protein or carbohydrate between CT and US males (**Table 2**). However, in females there was a significant difference in the linear effects of protein and carbohydrate on lifespan across the selection regimes (**Table 2**). This suggests that the effects of both nutrients were stronger in CT females (i.e., the linear gradients were steeper for both protein and carbohydrate, meaning that lifespan increased more with each additional unit of macronutrient consumed).

**Table 2:** Comparison of nutritional landscapes for lifespan between sexes and lines of *C. cosyra* selected on age of female oviposition.

Lifespan comparison	SSr	SSc	DF1	DF2	F	p value
CT vs US males						
Linear	195.573	194.732	2	592	1.28	0.279
Quadratic	161.661	161.147	2	588	0.93	0.392
Correlational	155.836	155.815	1	586	0.08	0.779
CT vs US females						
Linear	168.699	166.744	2	592	3.47	**0.032^A^**
Quadratic	143.904	142.843	2	588	2.18	0.113
Correlational	139.673	139.558	1	586	0.48	0.487
CT females vs CT males						
Linear	133.874	130.844	2	592	5.95	**0.003^B^**
Quadratic	100.026	98.955	2	588	3.18	**0.042**
Correlational	94.123	94.113	1	586	0.03	0.968
US females vs US males						
Linear	232.207	230.442	2	592	2.26	0.104
Quadratic	206.340	205.036	2	588	1.87	0.155
Correlational	201.366	201.260	1	586	0.31	0.578

Univariate tests: ^A^ Protein: χ2 = 6.87, p = 0.008; Carbohydrate: χ2 = 972.6, p < 0.001. ^B^ Protein: χ2 = 42.05, p < 0.001; Carbohydrate: χ2 = 2121.6, p < 0.001.

The analyses were run on results obtained from Experiment I where diets were provided under no-choice conditions.Bold values indicate significant effects (p<0.05).

Having compared nutrient landscapes for lifespan between selection lines within each sex, we then compared landscapes between females and males belonging to the same selection lines. We did not find differences in the linear, quadratic or correlational effects of protein and carbohydrate across the sexes in US lines (**Table 2**). However, CT females and males differed in their linear and quadratic effects for protein and carbohydrate (**Table 2**). The difference in linear effects is due to protein and carbohydrate contributing more to lifespan expression in CT females than males, and the difference in the quadratic effect is due to a significant quadratic effect of protein in females but not in males.

When comparing lifespan to reproductive traits in CT females we found differences in the linear effects for all comparisons and in quadratic effects when lifespan was compared to daily egg production (**Table 3**). The linear differences between lifespan and lifetime egg production in CT females arose because protein and, to a greater extent, carbohydrate made a greater contribution to the expression of lifespan than lifetime egg production (**Table 1**). The significant difference in the linear effects between daily egg production and lifespan in CT females was due to a difference in the magnitude of the contribution of carbohydrate to the expression of these traits, and to a lesser extent to the difference in protein contribution (**Table 1**). The significant difference in the quadratic effects reflects the absence of a quadratic effect of carbohydrate in daily egg production but not lifespan, and a difference in the direction of the quadratic effect of protein (peak for daily egg production, through for lifespan) (**Table 1**). In US females we detected significant differences in the linear effects of nutrients when comparing lifespan to lifetime egg production and daily egg production (**Table 3**), which were caused only by a stronger contribution of carbohydrate to lifespan.

**Table 3:** Comparison of nutritional landscapes between lifespan (LS) and female reproductive traits in *C. cosyra* lines selected on age of oviposition.

Female traits comparison	SSr	SSc	DF1	DF2	F	p value
CT LS vs LEP						
Linear	253.075	233.164	2	592	25.36	**< 0.001^A^**
Quadratic	208.291	207.023	2	588	1.68	0.185
Correlational	203.721	203.662	1	586	0.17	0.680
CT LS vs DEP						
Linear	365.359	289.810	2	592	77.42	**< 0.001^B^**
Quadratic	269.838	264.171	2	588	6.33	**0.002**
Correlational	262.291	261.803	1	586	1.09	0.296
US LS vs LEP						
Linear	368.947	348.399	2	592	17.51	**< 0.001^C^**
Quadratic	341.674	339.914	2	588	0.22	0.212
Correlational	339.586	338.643	1	586	1.63	0.201
US LS vs DEP						
Linear	510.462	431.885	2	592	54.16	**< 0.001^D^**
Quadratic	426.121	423.423	2	588	1.88	0.153
Correlational	423.369	421.649	1	586	2.39	0.122
CT LEP vs DEP						
Linear	403.784	384.839	2	592	14.57	**< 0.001^E^**
Quadratic	366.319	364.754	2	588	1.26	0.284
Correlational	363.459	363.254	1	586	0.32	0.570
US LEP vs DEP						
Linear	585.446	565.531	2	592	10.42	**< 0.001^F^**
Quadratic	564.104	563.486	2	588	0.32	0.724
Correlational	562.886	562.798	1	586	0.09	0.762
CT LEP vs US LEP						
Linear	422.634	419.920	2	592	1.91	0.148
Quadratic	412.085	409.748	2	588	1.67	0.187
Correlational	409.464	408.449	1	586	1.45	0.228
CT DEP vs US DEP						
Linear	561.956	557.085	2	592	2.59	0.076
Quadratic	550.326	548.697	2	588	0.92	0.396
Correlational	548.632	547.901	1	586	0.78	0.286

Univariate tests: ^A^ Protein: χ2 = 81.41, p < 0.001; Carbohydrate: χ2 = 909.05, p < 0.001. ^B^ Protein: χ2 = 60.44, p < 0.001; Carbohydrate: χ2 = 350.69, p < 0.001. C Protein: χ2 = 0.06, p =0.803; Carbohydrate: χ2 = 251.18, p < 0.001. ^D^ Protein: χ2 = 0.62, p =0.428; Carbohydrate: χ2 = 77.78, p < 0.001. ^E^ Protein: χ2 = 70.60, p < 0.001; Carbohydrate: χ2 = 154.5, p < 0.001. F Protein: χ2 = 8.90, p =0.003; Carbohydrate: χ2 = 14.07, p < 0.001.

The analyses were run on results obtained from Experiment I where diets were provided under no-choice conditions. Bold values indicate significant effects (p<0.05).

Surface responses for lifetime egg production and daily egg production differed in their linear effects for both CT and US lines (**Table 3**). Both nutrients were responsible for this significant difference (**Table 3**), and this is the result of the larger contribution of carbohydrate towards the expression of lifetime egg production than daily egg production (**Table 1**). When comparing surface responses for reproductive traits across selection lines we did not detect any significant differences (**Table 3**).

**Figure 3:**
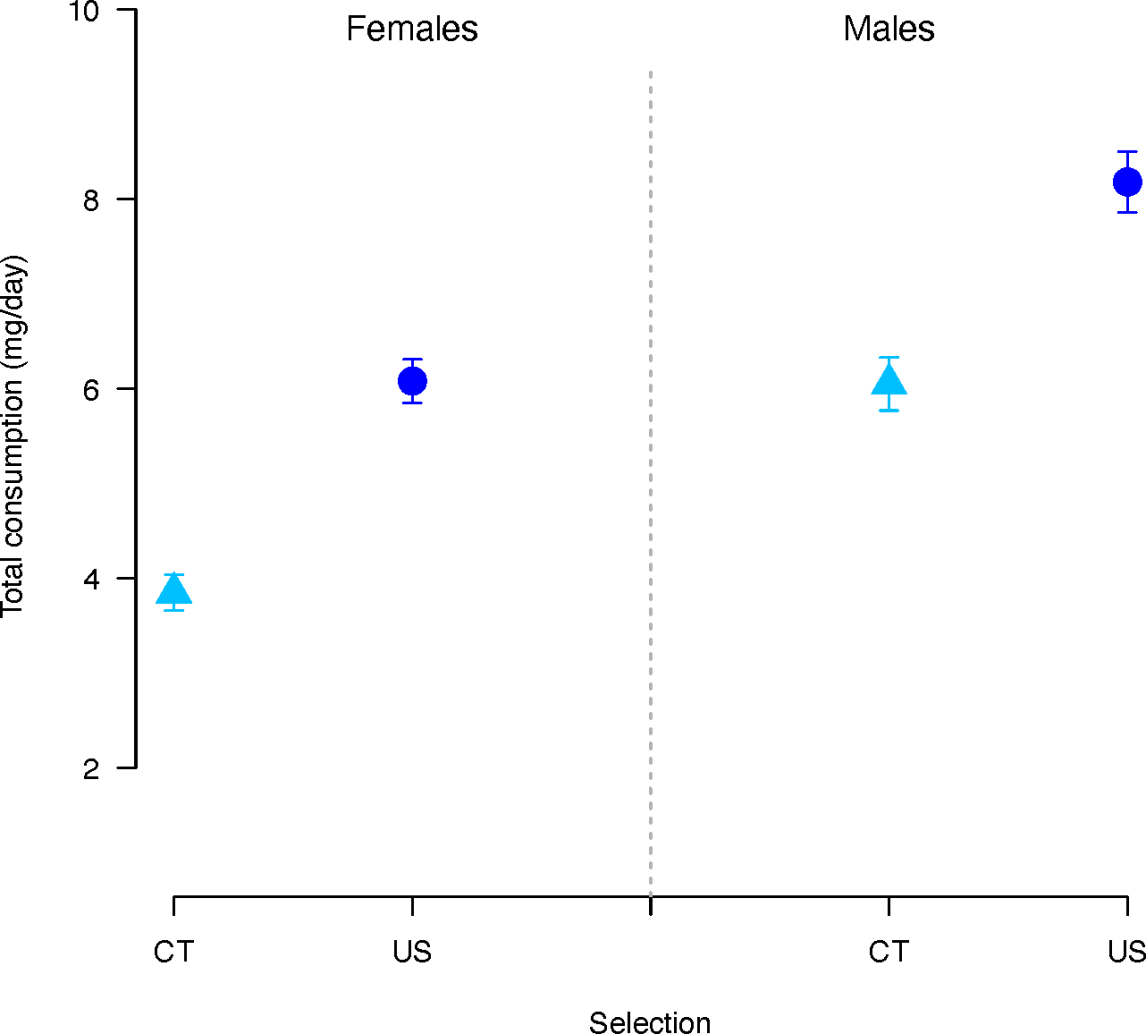
Total nutrient consumption (P+C) in females and males *C. cosyra* of control (light blue triangles) and upward-selected (blue circles) lines on age of female oviposition. Error bars indicate the standard error of the mean. Each bar represents 300 individuals.

Total consumption (protein and carbohydrate together) was significantly affected by selection regime (χ2 = 51.09, p < 0.001) and sex (χ2 = 35.53, p < 0.001). Overall, CT flies consumed significantly less diet than US flies (estimate = -0.56, p < 0.001) (**Figure 3**), and males significantly more than females (estimate = 0.36, p < 0.001). There was also a significant effect of the concentration and the P:C ratio (Concentration: χ2 = 6.86, p = 0.008; P:C ratio: χ2 = 347.28, p < 0.001). Consumption decreased as the concentration (coefficient= 0.004, p = 0.008) and P:C ratio (coefficient = -1.14, p < 0.001) increased.

### Experiment II: nutrient intake under dietary choice

**Figure 4:**
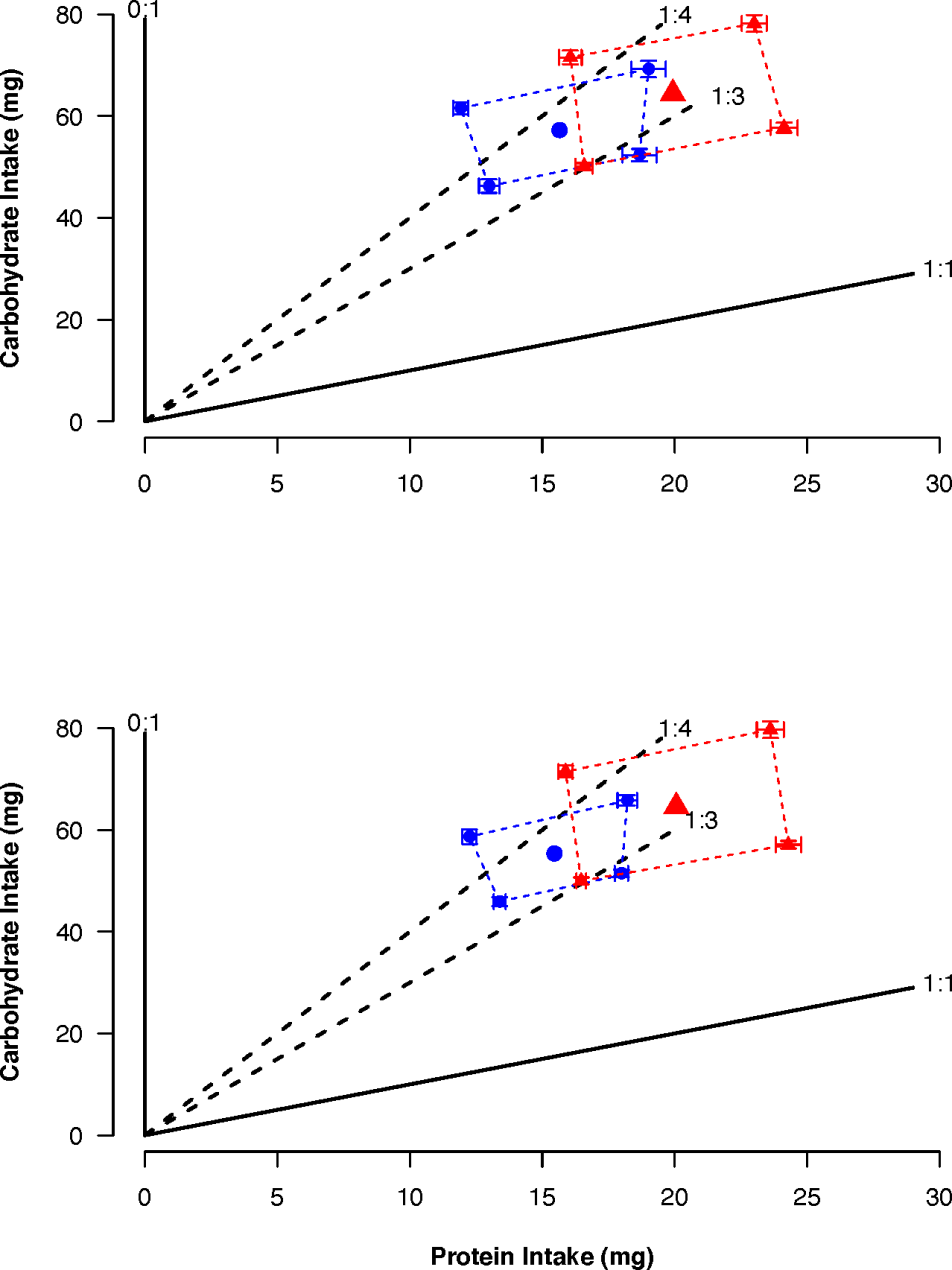
Average total intake ( ± SE) of protein and carbohydrate when females (top) and males (bottom) of control (blue) and upward-selected (red) lines were given the choice between two diets over a 16 day feeding period. The regulated intake points are represented by the large blue circles and red triangles. Diet pairs were: Pair 1: 1:1 (180 g/L) vs 0:1 (180 g/L); Pair 2: 1:1 (180 g/L) vs 0:1 (360 g/L); Pair 3: 1:1 (360 g/L) vs 0:1 (180 g/L); Pair 4: 1:1 (360 g/L) vs 0:1 (360 g/L). For each sex and pair, blue circles and triangles represent 20 individuals and red circles and triangles 15 individuals.

The total amount of nutrients consumed by females and males was similar within selected lines but differed between lines (**CT**: female: Protein = 15.65 mg, Carbohydrate = 57.33 mg; male: Protein = 15.46 mg, Carbohydrate = 55.45 mg; **US**: female: Protein = 19.93 mg, Carbohydrate = 64.39 mg; male: Protein = 20.06 mg, Carbohydrate = 64.55 mg). Despite the difference between selected lines in the amount of nutrients consumed, the regulated intake points for the CT and US lines were in the same region of the nutrient space between the nutritional rails representing the P:C ratios 1:4 and 1:3 (**CT**: 1:3.6; **US**: 1:3.2) and were the same between sexes (**Figure 4**).

We found that flies only ate at random when feeding on 1:1 (180 g/L) vs 0:1 (360 g/L) (Pair 2) for CT flies and 1:1 (180 g/L) vs 0:1 (180 g/L) (Pair 1) for US flies, and flies were not eating at random from each other diet pair (**Table S4**). The intake of carbohydrate differed between selected lines (**Table 4**). This was because CT flies had a lower intake than US ones (coefficient = -0.20, p = 0.041) (**Figure 4**). In addition, there was a significant effect of the protein intake (**Table 4**), as intake of carbohydrate increased with the intake of protein (coefficient = 0.01, p < 0.001). The absence of a significant interaction between protein intake and selection regime indicated that the regulated intake point (i.e., self-selected P:C ratio) did not differ between selected lines. We found no difference between strategies of nutrient regulation across the sexes within the same selected lines (**Table 4**). In both selected lines, there was a significant effect of the protein intake on the carbohydrate intake (**Table 4**). The intake of carbohydrate increased with the intake of protein (CT: coefficient = 0.02, p < 0.001; US: coefficient = 0.01, p = 0.016).

**Table 4:** Comparison of the regulated intake of carbohydrate by *C. cosyra* between lines selected on age of female reproduction and sexes.

	χ2	df	p
Selection			
Protein	15.91	1	**< 0.001**
Selection	4.17	1	**0.041**
Selection x Protein	2.32	1	0.127
CT: female *vs* male			
Protein	29.39	1	**< 0.001**
Sex	0.01	1	0.918
Sex x Protein	0.02	1	0.891
US: female *vs* male			
Protein	5.78	1	**0.016**
Sex	0.01	1	0.981
Sex x Protein	0.01	1	0.983

The analyses were run on results obtained from Experiment II where flies were able to choose between a pair of diets varying in the P:C ratio and/or concentration. Bold values indicate significant effects (p<0.05).

## Discussion

In insects, the trade-off between lifespan and reproduction is often mediated by diet in one or both sexes (13, 16, 21, 24, 34, 35). These studies have also reported that dietary optima for reproductive traits and lifespan are sex specific and that females often have different dietary optima for lifespan and reproduction. Given that optimal foraging theory predicts that individuals regulate intake to optimize fitness (36), this means that optimizing fitness *via* nutrient regulation may involve a compromise between consuming a diet that is optimal for lifespan and one that promotes reproduction. In keeping with this idea, female insects tend to regulate towards an intake that is not optimal for either lifespan or daily reproductive effort, but rather lies between the two divergent optima (13, 21, 37). Therefore, in our upward-selected flies where oviposition substrate was available at a later age, which induced a drop in reproductive effort and lifespan (25), we expected the amount or blend of nutrients ingested to differ from those of the control flies, towards a nutrient blend that promoted survival until the reproduction substrate became available.

The selection regime had a minor impact on how both nutrients affected lifespan. Both nutrients contributed slightly more to the expression of lifespan in control than in upward-selected lines. Nevertheless, in both lines and both sexes, carbohydrate was the most important nutrient for lifespan, with the trait peaking at low protein to high carbohydrate ratios, and high intake of diet. This aligns with previous studies on other insect species and *C. cosyra* (15, 20). In contrast to lifespan, the effects of nutrition on reproductive traits differed between control and upward-selected flies. Lifetime egg production increased with the intake of both nutrients, but the contribution of protein and carbohydrate was greater in control than upward-selected flies. Differences in how nutrition affected phenotype were most pronounced for daily egg production, with an absence of peak in upward-selected lines but a peak at high P:C ratios in control flies. While both carbohydrate and protein contributed equally to the expression of daily egg production in control lines, only protein contributed to daily egg production in upward-selected ones. Thus, both laboratory adapted lines differed from non-laboratory adapted flies (assayed in a previous study) in which both macronutrients contributed to optimal expression of female reproductive traits (15).

In the control lines, the magnitude of the nutrients’ contribution to the phenotype differed between sexes, but both macronutrients were required to optimize lifespan expression. This was not the case in upward-selected lines. Carbohydrate intake alone modulated lifespan expression in upward-selected males, but in females, lifespan was positively correlated with the intake of both nutrients (although the contribution of carbohydrate was tenfold greater than the contribution of protein). These differences are in contrast with a previous study using non laboratory adapted *C. cosyra* (15), where protein was detrimental to lifespan, and the magnitude of this deleterious effect was greater in males than females. Here, those differences occurred regardless of the selection regime, and therefore the differences between Malod et al. (15) and the current study might have been caused by laboratory adaptation of the fly colonies. In addition, while the proportion of protein and carbohydrate ingested by the laboratory adapted lines from this study were similar to those of wild flies (15), there was a noticeable change in nutrient preference across the two studies. Wild marula flies show a clear preference for protein over carbohydrate (15), whereas this pattern has been lost in both lines of the current study. Instead, control flies displayed a preference for carbohydrate over protein, and upward-selected flies rather preferred carbohydrate or had no preference. The increase in lifespan and total number of eggs laid by control females in comparison with wild females from Malod et al. (15) also suggests that there was laboratory adaptation. This is similar to observations in *Bactrocera tryoni*, where females from a long-term laboratory strain produced more eggs than wild females (38). Fruit flies rely on amino acids as a source of nitrogen (39), and given that in the wild such resources are difficult to acquire for fruit flies, it was suggested in Malod et al. (15) that the preference for protein in wild flies is an adaptation to resource scarcity. In an environment where protein is not limited, the selection pressure for protein preference may have been relaxed in favour of carbohydrate. This aligns with observations in *Blatella germanica*, where preference for protein in wild cockroaches is higher than in laboratory cultured ones (40).

Nutrient regulation did not differ between selected lines. Both lines regulated their nutrient intake similarly, at 1:3.6 P:C and 1:3.2 P:C for control and upward-selected flies respectively. This regulated intake point is nearly identical (1:3 P:C) to that found for non-laboratory adapted marula flies (15), and another species of the same family, *Bactrocera tryoni* (37). While this ratio allows *C. cosyra* or *B. tryoni* to maximise female reproductive effort, or at least get close to the optimum, with a mild to moderate cost to lifespan, this is not the case in our laboratory-selected lines. Although this ratio also allows the control flies to perform well, they do not require consumption of such a protein-biased ratio to optimise reproductive effort, which was as good on lower P:C ratios. Reproductive traits in control females can be optimised on several P:C ratios if nutrient intake is high. However, this is not the case for lifespan as it peaks at low P:C ratios with high nutrient intake. Eating towards a ratio between 1:4 and 1:3 P:C has a cost to lifespan but does not bring additional gains in reproductive effort for a control female. In the upward-selected flies, the situation is different as expression of their reproductive traits was poor and there was no peak for reproductive traits. Both selected lines ate towards a P:C ratio that has no benefit for reproductive trait expression and shortens lifespan. This may show that, despite changes in life-history strategies induced by a change in the environment (i.e., time of oviposition substrate availability), the proportion of protein and carbohydrate that an individual will target is well conserved at species level. This is even if it is no longer the best compromise in terms of lifespan or reproductive effort. This may mean that if dietary optima shift due to environmental change, organisms are not good at tracking their changing nutritional needs, as it was previously observed in African honey bees (41). An alternative explanation is that that other traits, which have not been considered in this study but are critical to fitness, benefit from this P:C ratio. That is, experimental insects were eating towards a nutrient blend that had a positive impact on other traits. One candidate is immunity, as there is a trade-off between reproduction and traits associated with immune functions (e.g. encapsulation ability in insects), and this trade-off appears to be mediated by diet (17).

Flies from upward-selected lines consistently ingested more nutrients than the control flies, whether they were in a no-choice or dietary choice situation. This is surprising, as analysis showed that trait expression increased with nutrient intake. Yet, despite consuming more nutrients, the fitness of the upward-selected flies was lower. Because both lines were fed the same diets, the increased diet ingestion observed in upward-selected flies cannot be attributed to compensatory feeding behaviours usually observed when insects are fed lower food quality (13, 37, 42). On the nutritional landscapes it appears that, while they ate more than the control flies, they never consumed enough nutrients to reach similar trait expression. It could be that the selection regime impaired nutrient assimilation in the upward-selected flies. Assimilation and nutrient allocation are essential to a good expression of life-history traits, and physiological dysfunctions in nutrient assimilation can lead to early death (43). Gut bacteria are essential to fruit fly nutrition and laboratory adaptation is known to alter gut microbiome diversity with potential deleterious consequences on fitness and life-history traits such as lifespan and reproduction (44–47). Therefore, it might be that selecting upwards on age of female reproduction negatively affected the gut microbiome. This demonstrates a more general theme, that to really understand the relationship between nutrition and phenotype requires also considering what happens after nutrient ingestion. For example, it was shown that egg resorption may be one means of salvaging nutrients when individuals consume a suboptimal nutrient blend (48). Although it is not known if *C. cosyra* can resorb their eggs, apoptosis has been observed in a closely related species, *C. capitata* (49), meaning that *C. cosyra* is potentially equipped for egg resorption. Furthermore, females from the *Bactrocera* genus reabsorb their follicles when the environment is unsuitable (e.g., temperature or absence of host), as a possible means of nutrient reallocation towards survival (50, 51). This may be one means by which female *C. cosyra* buffer the impacts of consuming nutritionally imbalanced or inadequate foods.

These data demonstrate how a change in reproductive scheduling induced by environmental variation in resources acts on nutrient requirements and regulation. The environment we created, with a substrate for reproduction available at different ages between selected lines, did not affect nutritional requirements. In a world where environmental conditions are changing and host plant availability may shift, this suggests that nutritional requirements for herbivorous insects, such as *C. cosyra*, do not necessarily need to evolve. Furthermore, the amount of nutrient ingested was higher in upward-selected flies, although they had an overall fitness lower than flies that were not selected upwards. We suggest that this higher food intake might be a behavioural response to physiological dysfunctions caused by laboratory adaptation, but it might also be that the higher nutrient intake is necessary to meet nutrient requirements in other life-history traits that we have not measured. Further investigation is needed to understand how life-history strategies may affect nutrition. For example, downward-selection for age of female reproduction (i.e., an environment with an oviposition substrate more frequently available) in the marula fly reduces lifespan and improves early reproductive effort (25). Therefore, investigations using other types of selection pressure while measuring more life-history traits may bring valuable information on how well conserved the relationship between ingested nutrients and trait expression is and how it connects to temporal fluctuations of the environment.

## Data availability statement

The raw data supporting the conclusions of this article will be made available by the authors, without undue reservation.

## Author contributions

CA, CW and SN conceived the ideas and designed methodology. KM and JH conducted data analysis. KM collected laboratory data. KM led the writing of the manuscript. All authors contributed to the article and approved the submitted version.
