# Supplementary material for: Selection on female reproductive schedules in the marula fly, Ceratitis cosyra (Diptera: Tephritidae) affects dietary optima for female reproductive traits but not lifespan
Source: Front Insect Sci. 2023 May 17;3:1166753. doi: 10.3389/finsc.2023.1166753 (PMC10926420; doi:10.3389/finsc.2023.1166753)
Supplement: Supplementary file 1 [file DataSheet_1.docx]

**Selection on female reproductive schedules in the marula fly, *Ceratitis cosyra* (Tephritidae) affects dietary optima for female reproductive traits but not lifespan.**

**Kevin Malod^1*^, C. Ruth Archer^2^, John Hunt^3^, Susan W. Nicolson^1^, Christopher W. Weldon^1^.**

Table S1. Experimental diets fed to female and male *C. cosyra* in either no-choice or choice experiment.

| P:C | Concentration (g/L) | Sucrose (g/L) | Amino Acids (g/L) | Vitamin (g/L) | Cholesterol (g/L) | RNA (g/L) | Wesson salt (g/L) |
| --- | --- | --- | --- | --- | --- | --- | --- |
| 0:1 | 360 | 360.00 | 0.00 | 3.6 | 4.0 | 10.0 | 10.0 |
|  | 180 | 180.00 | 0.00 | 3.6 | 4.0 | 10.0 | 10.0 |
|  | 45 | 45.00 | 0.00 | 3.6 | 4.0 | 10.0 | 10.0 |
| 1:8 | 360 | 320.00 | 40.00 | 3.6 | 4.0 | 10.0 | 10.0 |
|  | 180 | 160.00 | 20.00 | 3.6 | 4.0 | 10.0 | 10.0 |
|  | 45 | 40.00 | 5.00 | 3.6 | 4.0 | 10.0 | 10.0 |
| 1:4 | 360 | 288.00 | 72.00 | 3.6 | 4.0 | 10.0 | 10.0 |
|  | 180 | 144.00 | 36.00 | 3.6 | 4.0 | 10.0 | 10.0 |
|  | 45 | 36.00 | 9.00 | 3.6 | 4.0 | 10.0 | 10.0 |
| 1:2 | 360 | 240.00 | 120.00 | 3.6 | 4.0 | 10.0 | 10.0 |
|  | 180 | 120.00 | 60.00 | 3.6 | 4.0 | 10.0 | 10.0 |
|  | 45 | 30.00 | 15.00 | 3.6 | 4.0 | 10.0 | 10.0 |
| 1:1 | 360 | 180.00 | 180.00 | 3.6 | 4.0 | 10.0 | 10.0 |
|  | 180 | 90.00 | 90.00 | 3.6 | 4.0 | 10.0 | 10.0 |
|  | 45 | 22.50 | 22.50 | 3.6 | 4.0 | 10.0 | 10.0 |

**Table S2: Mixture of micronutrients incorporated into all experimental diets fed to *C. cosyra***

|  |  |  | g/(100g) | Producer (Product #) |
| --- | --- | --- | --- | --- |
| Amino acid mixture |  | l-Alanine | 4.87 | Merck (1.01007.0100) |
|  |  | l-Arginine | 6.67 | Sigma (11039) |
|  |  | l-Aspartic acid | 7.05 | Merck (1.00126.0100) |
|  |  | l-Cysteine | 2.55 | Merck (1.02839.0100) |
|  |  | l-Glutamic acid | 24.51 | Merck (8.14632.0250) |
|  |  | Glycine | 5.62 | Merck (1.04201.0100) |
|  |  | l-Histidine | 2.85 | Merck (1.04351.0100) |
|  |  | l-Isoleucine | 3.52 | Merck (1.05362.0100) |
|  |  | l-Leucine | 6.75 | Merck (1.05360.0250) |
|  |  | l-Lysine monohydrochloride | 3.67 | Merck (1.05700.0100) |
|  |  | l-Methionine | 1.72 | Merck (1.05707.0025) |
|  |  | l-Phenylalanine | 4.42 | Merck (1.07256.0100) |
|  |  | l-Proline | 7.80 | Merck (5370-GM) |
|  |  | l-Serine | 4.87 | Merck (1.07769.0100) |
|  |  | l-Threonine | 3.37 | Merck (1.08411.0100) |
|  |  | l-Tryptophan | 1.80 | Merck (1.08374.0010) |
|  |  | l-Tyrosine | 3.00 | Merck (1.08371.0025) |
|  |  | l-Valine | 4.95 | Merck (1.08495.0100) |
| Cholesterol |  |  | 100 | Sigma (C3045) |
| RNA |  |  | 100 | Sigma (R6625) |
| Sucrose |  |  | 100 | Merck (573113) |
| Vanderzant vitamin mixture |  | Ascorbic acid | 27.00 | Sigma (V1007) |
|  |  | Biotin | 0.002 |  |
|  |  | Choline chloride | 5.00 |  |
|  |  | Folic acid | 0.03 |  |
|  |  | Glucose | 64.70 |  |
|  |  | Inositol | 2.00 |  |
|  |  | Niacinamide | 0.10 |  |
|  |  | Pantothenic acid hemicalcium | 0.10 |  |
|  |  | Pyridoxine HCl | 0.03 |  |
|  |  | Riboflavin | 0.05 |  |
|  |  | Thiamin HCl | 0.03 |  |
|  |  | Tocopherol type VI | 0.80 |  |
|  |  | Vitamin B12 | 0.20 |  |
| Wesson salt mixture |  | Calcium carbonate | 21.00 | Sigma |
|  |  | Copper sulfate | 0.04 |  |
|  |  | Ferric phosphate | 1.47 |  |
|  |  | Magnesium sulfate | 9.00 |  |
|  |  | Manganese sulfate | 0.02 |  |
|  |  | Potassium aluminium sulfate | 0.01 |  |
|  |  | Potassium chloride | 12.00 |  |
|  |  | Potassium iodide | 0.005 |  |
|  |  | Potassium phosphate monobasic | 31.00 |  |
|  |  | Sodium chloride | 10.50 |  |
|  |  | Sodium fluoride | 0.06 |  |
|  |  | Tricalcium phosphate | 14.90 |  |
| Nipagin |  |  | 1.5 | Sigma (H3647) |

**Figure S1. Standard curve to estimate volume from length. I filled pipette tips with a volume ranging from 2 µL to 120 µL and measured the length (mm) at 2 µL intervals. For each step, 13 measures were taken.**


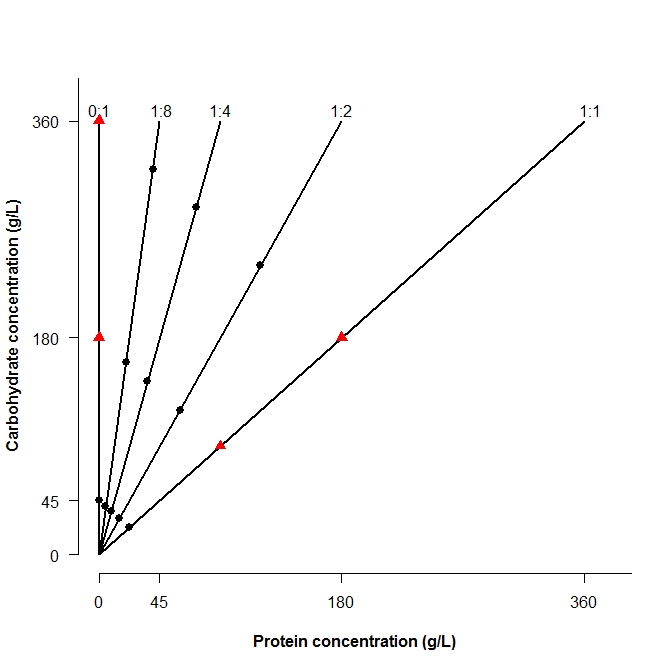


**Figure S2. Nutrient content and concentration of the diets provided in the dietary choice experiment. The red triangles indicate the position in the nutrient space of the four diets used to form dietary pairs.**

**Experiment II statistical analyses:**

We calculated the intake of P and C expected if flies fed at random. From the observed intake of both nutrients, we subtracted the expected intake, and the difference was compared with zero (one sample t-tests). Within each pair, values not significantly different from zero indicate that individuals ate randomly on the given diet pair. To determine the preferred diet across selection regime and sex, we used a paired t-test to compare intakes within each diet pair.

**Table S3. *Post hoc* pairwise comparisons of the estimated marginal means between diet pairs provided during the choice experiment to CT and US flies.** Diet pairs were: Pair 1: 1:1 (180 g/L) vs 0:1 (180 g/L); Pair 2: 1:1 (180 g/L) vs 0:1 (360 g/L); Pair 3: 1:1 (360 g/L) vs 0:1 (180 g/L); Pair 4: 1:1 (360 g/L) vs 0:1 (360 g/L). The values indicated are the estimates and p values.

|  | Pair 1 | Pair 2 | Pair 3 | Pair 4 |
| --- | --- | --- | --- | --- |
| Protein | | | | |
| Pair 1 |  | **0.06 / p = 0.027** | **-0.35 / p < 0.001** |  |
| Pair 2 |  |  |  |  |
| Pair 3 |  | **0.41 / p < 0.001** |  |  |
| Pair 4 | **0.34 / p < 0.001** | **0.40 / p < 0.001** | -0.01 / p = 0.737 |  |
| Carbohydrate | | | | |
| Pair 1 |  | **-0.31 / p < 0.001** | **-0.13 / p < 0.001** |  |
| Pair 2 |  |  |  |  |
| Pair 3 |  | **-0.18 / p < 0.001** |  |  |
| Pair 4 | **0.41 / p < 0.001** | **0.11 / p < 0.001** | **0.29 / p < 0.001** |  |

Comparison of the difference between observed and expected intake of P and C to a mean of zero revealed that the randomness of the intake was selection and diet pair dependent. Females and males of the CT flies were not consuming nutrients at random in some diet pairs (1:1 (180 g/L) vs 0:1 (180 g/L); 1:1 (360 g/L) vs 0:1 (360 g/L)) (Table S4). However, both sexes were feeding at random on one diet pair (1:1 (180 g/L) vs 0:1 (360 g/L)). In contrast, females and males from the US line were eating at random when the diet pairing was (1:1 (180 g/L) vs 0:1 (180 g/L)), and were regulating their nutrients intake on diet pairing (180 g/L) vs 0:1 (360 g/L) and 1:1 (360 g/L) vs 0:1 (360 g/L) (Table 3.8) (Fig. 4).

**Table S4. Comparisons to zero of the difference between observed intake and expected intake using one sample T-tests in female (F) and male (M) of control (CT) and upward selected (US) lines.** Pair 1: 1:1 (180 g/L) vs 0:1 (180 g/L); Pair 2: 1:1 (180 g/L) vs 0:1 (360 g/L); Pair 3: 1:1 (360 g/L) vs 0:1 (180 g/L); Pair 4: 1:1 (360 g/L) vs 0:1 (360 g/L). A positive significant value for t indicates that individuals are not eating at random and prefer this nutrient. A negative value for t indicates that individuals are eating less than expected if they were feeding at random. NAs indicate that the observed and expected intake were exactly the same.

| **Line** | **Sex** | **Pair** | **Nutrient** | **t** | **p value** |
| --- | --- | --- | --- | --- | --- |
| **CT** | F | 1 | P | -8.66 | **<0.001** |
|  |  |  | C | 8.66 | **<0.001** |
|  |  | 2 | P | -1.67 | 0.11 |
|  |  |  | C | 1.67 | 0.11 |
|  |  | 3 | P | -21.02 | **<0.001** |
|  |  |  | C | NA | NA |
|  |  | 4 | P | -9.2 | **<0.001** |
|  |  |  | C | 9.2 | **<0.001** |
|  | M | 1 | P | -8.77 | **<0.001** |
|  |  |  | C | 8.77 | **<0.001** |
|  |  | 2 | P | 1.98 | 0.06 |
|  |  |  | C | -1.98 | 0.06 |
|  |  | 3 | P | -24.06 | **<0.001** |
|  |  |  | C | NA | NA |
|  |  | 4 | P | -10.09 | **<0.001** |
|  |  |  | C | 10.09 | **<0.001** |
| **US** | F | 1 | P | -0.58 | 0.56 |
|  |  |  | C | 0.58 | 0.56 |
|  |  | 2 | P | 6.83 | **<0.001** |
|  |  |  | C | -6.83 | **<0.001** |
|  |  | 3 | P | -15.06 | **<0.001** |
|  |  |  | C | NA | NA |
|  |  | 4 | P | -9.35 | **<0.001** |
|  |  |  | C | 9.35 | **<0.001** |
|  | M | 1 | P | -1.07 | 0.3 |
|  |  |  | C | 1.07 | 0.3 |
|  |  | 2 | P | 9.27 | **<0.001** |
|  |  |  | C | -9.27 | **<0.001** |
|  |  | 3 | P | -9.39 | **<0.001** |
|  |  |  | C | NA | NA |
|  |  | 4 | P | -8.66 | **<0.001** |
|  |  |  | C | 8.66 | **<0.001** |

Preference for a diet was selection and sex specific. In the CT line, regardless of the concentration, overall both sexes preferred the diet with C only (Table S5). This was not the case for the males on diet pair 2 (1:1 (180 g/L) vs 0:1 (360 g/L)) where a slight but significant preference was observed for the diet containing P. The preference for a diet was more complex in the US line. Both sexes did not display any preference for a diet in diet pair 1 (1:1 (180 g/L) vs 0:1 (180 g/L)). In diet pair 2 (1:1 (180 g/L) vs 0:1 (360 g/L)) the diet containing P was preferred whereas in diet pair 3 (1:1 (360 g/L)) vs 0:1 (180 g/L)) and 4 (1:1 (360 g/L) vs 0:1 (360 g/L)), diets with C only were preferred (Table S5). The cumulative intake of P and C were similar between sexes but differed between selection regimes as flies from the US line consumed more than those of the CT line (Fig 4).

**Table S5. Diet intake comparison (Wilcoxon Signed Rank tests with Bonferroni correction) in female (F) and male (M) of control (CT) and upward-selected (US) lines.** Pair 1: 1:1 (180 g/L) vs 0:1 (180 g/L); Pair 2: 1:1 (180 g/L) vs 0:1 (360 g/L); Pair 3: 1:1 (360 g/L) vs 0:1 (180 g/L); Pair 4: 1:1 (360 g/L) vs 0:1 (360 g/L).

| **Line** | **Sex** | **Pair** | **V** | **p** | **Adjusted p** |
| --- | --- | --- | --- | --- | --- |
| **CT** | F | 1 | 0 | < 0.001 | **< 0.001** |
|  |  | 2 | 65 | < 0.001 | **0.035** |
|  |  | 3 | 0 | < 0.001 | **< 0.001** |
|  |  | 4 | 1 | < 0.001 | **< 0.001** |
|  | M | 1 | 1 | < 0.001 | **< 0.001** |
|  |  | 2 | 155 | 0.06 | **0.015** |
|  |  | 3 | 0 | < 0.001 | **< 0.001** |
|  |  | 4 | 0 | <0. 001 | **< 0.001** |
| **US** | F | 1 | 46 | 0.45 | 0.113 |
|  |  | 2 | 118 | 0.001 | **< 0.001** |
|  |  | 3 | 0 | < 0.001 | **< 0.001** |
|  |  | 4 | 0 | < 0.001 | **< 0.001** |
|  | M | 1 | 39 | 0.25 | 0.063 |
|  |  | 2 | 120 | < 0.001 | **< 0.001** |
|  |  | 3 | 0 | < 0.001 | **< 0.001** |
|  |  | 4 | 0 | < 0.001 | **< 0.001** |
